# Bootstrapping Automated Testing for RESTful Web Services

**DOI:** 10.1007/978-3-030-71500-7_3

**Published:** 2021-02-24

**Authors:** Yixiong Chen, Yang Yang, Zhanyao Lei, Mingyuan Xia, Zhengwei Qi

**Affiliations:** 8 Universidad Autónoma de Madrid, Madrid, Spain; 9 University of Twente, Enschede, The Netherlands; 10 Shanghai Jiao Tong University, Shanghai, China; 11 AppetizerIO, Shanghai, China

**Keywords:** Fuzz Testing, RESTful Web Service, Type Inference.

## Abstract

Modern RESTful services expose RESTful APIs to integrate with diversified applications. Most RESTful API parameters are weakly typed, which greatly increases the possible input value space. This poses difficulties for automated testing tools to generate effective test cases to reveal web service defects related to parameter validation. We call this phenomenon the type collapse problem. To remedy this problem, we introduce FET (Format-encoded Type) techniques, including the FET, the FET lattice, and the FET inference to model fine-grained information for API parameters. Enhanced by FET techniques, automated testing tools can generate targeted test cases. We demonstrate Leif, a trace-driven fuzzing tool, as a proof-of-concept implementation of FET techniques. Experiment results on 27 commercial services show that FET inference precisely captures documented parameter definitions, which helps Leif to discover 11 new bugs and reduce $72\% \sim 86\%$ fuzzing time as compared to state-of-the-art fuzzers.

## 1 Introduction

The REST (Representational State Transfer) architecture [28] nowadays has dominated the design of complex web services, such as public clouds (e.g. AWS and Azure), social networking (e.g. Facebook and Twitter), and code hosting (e.g. GitHub and GitLab). Typically, a RESTful web service exposes a set of RESTful APIs. A client requests an API providing parameter values, and the service responds with data represented in some common exchange format (e.g. JSON or XML). According to a recent survey of 40 real-world popular RESTful web services [36], modern services involve an average of 64 APIs and over 20 parameters per API. Testing such an input space of possible parameter value combinatorics is challenging, and therefore automated testing is indispensable.

Since RESTful APIs are intended for applications implemented by different programming languages, API parameters are weakly typed. An investigation on 27 RESTful web services [19] shows that over 67% of the parameters are

`string`-typed, about 32% are `number`-typed, and the remaining 1% are `boolean`-typed or `object`-typed. Overusing primitive data types significantly increases the possible input value space. For example, a `string`-typed parameter can take values varying from a specific URL to a comment about a YouTube video. This poses difficulties for generating effective test cases. Consequently, many automated REST testing tools are ineffective while RESTful web services suffer from various input-related attacks, such as integer overflow attacks and SQL injection attacks [18]. We call this phenomenon the *type collapse problem*.

The solution is to bridge the gap for automated testing tools to have a better understanding of parameters. We observe that though parameter types are weak, their values usually have distinct formats. For example, a datetime parameter may require an `ISO8601` date string. This motivates us to introduce the *FET (Format-encoded Type)* which combines *data types* and *value formats* to describe parameters in fine grains. For instance, the `SHA1` FET represents 40-digit-hex `string`-typed parameters. Furthermore, we introduce the FET lattice which hierarchically organizes a set of FETs by a partial order, along with the FET inference which seeks suitable FETs among a FET lattice for parameters in an unambiguous manner.

To manifest how to enhance automated REST testing by FET techniques, we implement Leif, a trace-driven fuzz testing tool. Leif gains fine-grained parameter information by performing FET inference on HTTP traffic and then mutates parameter values to mimic real attacks based on the inferred results. We apply Leif to real-world web services, and the experiment results are encouraging. FET techniques provide better bug-finding capability and bring 72% $\sim$ 86% fuzzing time reduction for Leif when compared to state-of-the-art fuzzing tools.

In particular, this paper makes the following contributions:

- We introduce FET techniques, including the FET, the FET lattice, and the FET inference, to remedy the type collapse problem and serve as a cornerstone for high-level automated testing tools.
- We implement Leif, a FET-enhanced fuzzing tool which showcases how to construct a ubiquitous FET lattice for common RESTful APIs and embed FET techniques in an existing testing workflow.
- We evaluate the accuracy of FET inference, and the result is encouraging (67% exact matches, 32% partial matches, and 1% mismatches on average).
- We evaluate Leif's bug-finding capability (11 distinct bugs detected in 27 commercial web services) as well as its testing efficiency (72% $\sim$ 86% fuzzing time reduction as compared to existing fuzzing tools).

The remainder of the paper is organized as follows. Section 2 analyzes the type collapse problem in detail. Section 3 introduces FET techniques to solve the type collapse problem. Section 4 introduces Leif as a proof-of-concept implementation of FET techniques. Section 5 presents the evaluation of FET techniques and Leif. Section 6 discusses related works and Section 7 concludes.

## 2 Motivation

It is essential for automated REST testing tools to generate test cases by filling parameters with automatically generated values. This procedure requires adequate information about parameters. Otherwise, the possible candidate space would become enormous even for one single parameter. Therefore, a majority of state-of-the-art automated testing tools focus on reducing the candidate space by sophisticated methodologies. For instance, RESTler [13] arranges multiple APIs in the producer-consumer order, and uses response data gained from the previous APIs to request the next. Chizpurfle [23] and EvoMaster [12] generate optimal candidate values based on evolutionary algorithms.

Nevertheless, the previous works have not focused on the root cause of the candidate space explosion. Since most RESTful APIs are designed for exchanging data between programs implemented by different languages (e.g., Java for mobile applications while Python for the service), only a few common *primitive data types* can be used to represent API parameters. For example, Amazon's online shopping web service takes about 2,400 parameters, among which 748 are `number`-typed (31%) and 1,581 are `string`-typed (66%) [19]. That is, types, which are supposed to be diversified, now collapse into very limited cases. Consequently, existing automated testing tools encounter a huge candidate space, e.g., solely knowing a parameter is `string`-typed spans a boundless candidate space from paragraphs of Shakespeare to specific datetime strings. In addition, it is difficult to pick up effective values that can pass parameter checking, then reach actual business logic, and finally trigger bugs. Figure 1 shows a code sample of a RESTful API (requires four parameters: `string`-typed `start`, `string`-typed `end`, `number`-typed `amount`, and `number`-typed `interest`). In order to generate an effective value which can reach business logic for the parameter `start`, a testing tool has to know it is an `ISO8601` datetime string. Unfortunately, since parameters are mainly in primitive data types, this information is usually hard to obtain. Therefore, the testing tool may treat it as an ordinary string and generate arbitrary strings which are all rejected by the parameter checking and thus are basically useless.

```python
def calculate_monthly_installment():
    try:
        start = parse(request.get("start"), "YYYY-MM-DDTHH:MM:SSZ")
        end = parse(request.get("end"), "YYYY-MM-DDTHH:MM:SSZ")
        amount = float(request.get("amount"))
        interest = float(request.get("interest"))
    except Exception:
        return make_response("Invalid Parameter", 400, "Bad Request")
    # business logic
    ...
```

**Fig. 1.** A Code Sample of a RESTful API (Written in Python).

The type collapse problem is the major obstacle to obtaining adequate parameter information and leads to inefficient automated testing. Therefore, our solution is to provide a fine-grained description method for parameters by exploiting both its data type and its value format. Leveraged by such information, we are able to bootstrap and enhance automated testing techniques to gain efficiency improvement when testing RESTful web services.

## 3 FET Techniques

To address the type collapse problem, we introduce FET techniques, including the FET (Format-encoded Type), the FET lattice, and the FET inference. A FET models an API parameter by its data type and its value format. A FET lattice hierarchically organizes a set of FETs based on a partial order. We design FET inference algorithms to seek suitable FETs among a FET lattice for parameters, and the inferred results are the critical information for bootstrapping test case generation strategies.

### 3.1 Type Lattice

The idea of the FET lattice is inspired by the type lattice [24] for programming languages widely used in compilation and program analysis [33,44,45]. A type lattice is a *complete lattice* defined on $\langle T, \sqsubseteq \rangle$, where $T$ is a set of data types (e.g. `long` in C/C++) and $\sqsubseteq$ is a partial order representing type convertibility. Every two lattice elements have a unique *least upper bound* and a unique *greatest lower bound*. An element $t_j$ is said to *cover* another element $t_i$ if and only if $t_i \sqsubset t_j$ but there does not exist a $t_m$ such that $t_i \sqsubset t_m \sqsubset t_j$, where $t_i \sqsubset t_j$ means $t_i \sqsubseteq t_j$ and $t_i \neq t_j$. Type lattices can model class inheritance hierarchies for object-oriented languages. In this context, for any two elements $t_i$ and $t_j$, $t_i \sqsubseteq t_j$ holds if and only if $t_i$ inherits from or equals to $t_j$. Figure 2 depicts a type lattice for `java.util.Collection` (each vertex represents a class or an interface, and each directed edge stands for the inheritance relationship).

The type lattice is the cornerstone of type systems for modern programming languages. In static compilation, the type lattice is applied to checking value assignment and type casting for code validity [38]. In dynamic compilation, e.g., JIT (Just-in-time Compilation) [14], it is employed to predict variable types at program points, so as to remove unnecessary type checking. The type lattice is a powerful tool to ensure the correctness and efficiency of programs. However, in the context of REST, API parameters only manifest limited primitive data types due to the type collapse problem, where the type lattice is no longer sufficient.

### 3.2 FET Lattice

A FET lattice is defined on $\langle \Psi \subseteq T \times F, \preceq \rangle$. A FET $\psi \in \Psi$ is defined by $(t_\psi, f_\psi)$, where $t_\psi \in T$ is a *data type*, and $f_\psi \in F$ is a *value format* or more specifically a *set* of values. $\preceq$ is a partial order that for any two FETs $\psi_i$ and $\psi_j$, $\psi_i \preceq \psi_j$

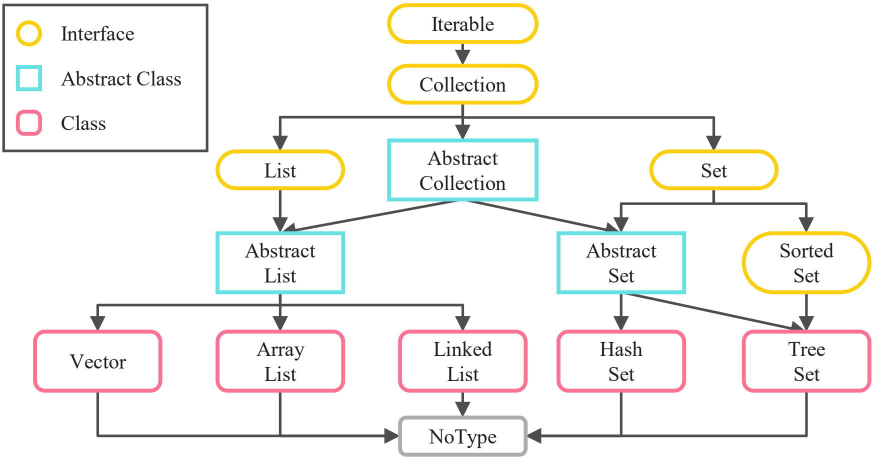

**Fig. 2.** A Type Lattice for the Java Collections Framework.

holds if and only if $t_{\psi_i}$ is *type-convertible* to $t_{\psi_j}$ and $f_{\psi_i}$ is a *subset* of $f_{\psi_j}$, denoted by $t_{\psi_i} \sqsubseteq t_{\psi_j}$ and $f_{\psi_i} \subseteq f_{\psi_j}$. A FET $\psi_i$ *covered* by $\psi_j$ implies that $\psi_i$ describes parameter features in a finer grain than $\psi_j$. $\psi_\top$ and $\psi_\bot$ are defined as ($\texttt{AnyType}, U$) and ($\texttt{NoType}, \emptyset$), where $U$ is the set containing arbitrary values. Figure 3 depicts an example FET lattice (a FET's name describes its value format, and FETs at the same level are identically colored).

**FET Acceptance for Parameter Values.** Similar to type lattices, FET lattices help to determine FETs for given parameter values. To achieve this, we define that a value $v$ is *accepted* by a FET $\psi$ if and only if $typeof(v) \sqsubseteq t_\psi$ and $v \in f_\psi$, denoted by $\psi \in acceptance(v)$. Otherwise $v$ is said to be *rejected* by $\psi$, denoted by $\psi \notin acceptance(v)$. Spontaneously, $\psi_\top$ accepts all values while $\psi_\bot$ accepts none. A value $v$ can be accepted by more than one FET, while the *greatest lower bound* of the acceptances describes the value in the finest grain. We call such an acceptance the *minimum acceptance* of $v$. The *predecessors* of the minimum acceptance accept $v$ but describe it in a coarser grain, while the *siblings* reject $v$ but describe other similar values in the same grain. The minimum acceptance, the predecessors, and the siblings of $v$ compose a *tree*, denoted by $\psi$-*tree*$(v)$. For example, for a `SHA1` string $v$, its minimum acceptance (the `SHA1` FET in Figure 3), the predecessors (`Hash`, `String`, and $\psi_\top$) and the siblings (`MD5`, and `SHA256`) compose the $\psi$-*tree*$(v)$.

**Avoiding the Ambiguity of FET Lattices.** As seen in Figure 3, if a single value is accepted by two sibling FETs (e.g. `MD5` and `SHA1`), the minimum acceptance will fall into the trivial $\psi_\bot$. Generally, a FET lattice is said to be *ambiguous* if there exist two FETs with the *same predecessor* can both accept the *same value*. To avoid ambiguity, a validation procedure is obligatory after a FET lattice is constructed, which is to ensure the value formats of every two sibling FETs with the same data type are always disjoint.

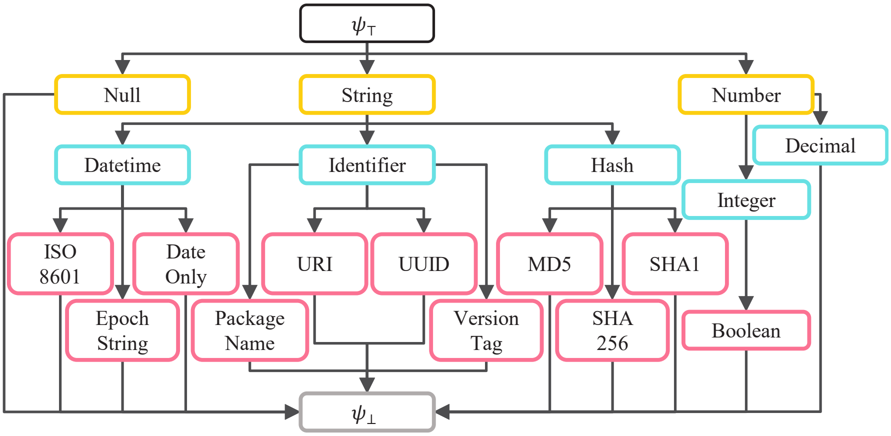

**Fig. 3.** An Example FET Lattice.

In practice, we specify value formats by the regular language, and provide a ubiquitous FET lattice [20] to model the most common RESTful parameters. We will elaborate FET lattice construction and verification in Section 4.2.

### 3.3 FET Inference

**Tree-merging FET Inference**. As discussed previously, for a single value $v$, a unique $\psi$-*tree*$(v)$ can always be found in an unambiguous FET lattice. A RESTful API parameter usually involves multiple values in practice. Hence we give the *tree-merging FET inference*. For a parameter with values $v_1, \cdots, v_n$, the tree-merging inference is to compute $\psi$-*tree*$(v_1), \cdots, \psi$-*tree*$(v_n)$, and then merge them into one tree. The merged tree is denoted by $\psi$-*tree*$^n(V_n)$ where $V_n = \{v_1, \cdots, v_n\}$. The tree-merging inference can be described as a "find-expand-merge" procedure: (1) find the minimum acceptance for a single value $v_i$ by performing a depth-first searching from $\psi_\top$ and add the predecessors along the searching path into the tree; (2) expand the tree by adding the siblings and then the $\psi$-*tree*$(v_i)$ is obtained; (3) repeat the step (1) and (2) for every value and merge all the trees. Step (1) and (2) are illustrated in Figure 4, and step (3) can be reduced to the DNS tree merging [25]. Assuming that the FET lattice has $l$ levels with $m$ FETs, the time complexity is $O(m)$ for computing one tree and $O(l)$ for merging two trees. Thus the time complexity of tree-merging FET inference for a parameter involving $n$ values is $O(n \cdot (m + l))$.

**Bitfield-boosting FET Inference**. In practice, we notice that the number of FETs $m$ in a lattice is a constant while the number of values $n$ is a variate (usually over 1,000). Therefore, we optimize the tree-merging FET inference based on three observations: (1) each FET can be uniquely represented by one bit in a $m$-bit bitfield, and therefore $\psi$-*trees* can be represented by several bits in such bitfields; (2) given a minimum acceptance, its $\psi$-*tree* can be uniquely

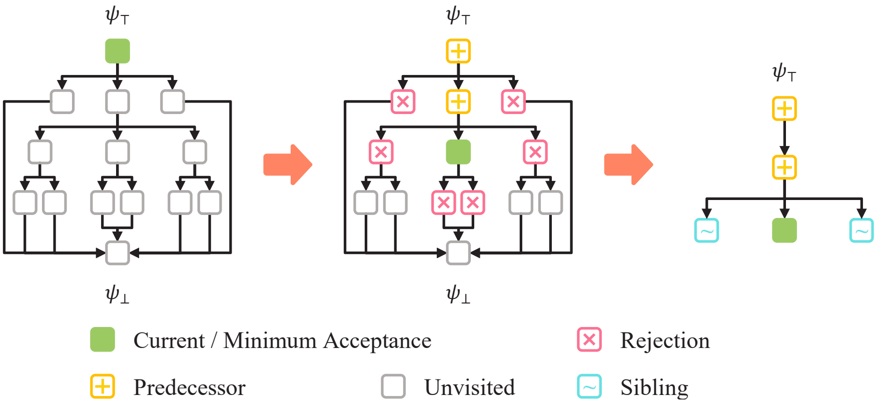

**Fig. 4.** Inferring $\psi$-*tree*$(v_i)$ for a Single Value $v_i$.

determined, so the $\psi$-*tree* for every FET can be computed before inference; (3) merging two $\psi$-*trees* is equivalent to performing a bitwise `OR` operation on their corresponding bitfields.

Hence, we give the *forward computation algorithm* and the *bitfield-boosting FET inference*. The forward computation traverses the lattice in breadth-first order, assigns a unique bitfield `ID` per FET, and computes the $\psi$-*tree*, as shown in Algorithm 1. Leveraged by the forward computation, the bitfield-boosting inference only needs to find the minimum acceptance by the depth-first searching, yields the bitfield `tree`, and merges it into the $\psi\text{-}tree^{i-1}(V_{i-1})$, as shown in Algorithm 2. Therefore, the $\psi\text{-}tree^{n}(V_n)$ can be efficiently computed by a series of bitwise `OR` operations instead of graph computations, reducing the time complexity from $O(n \cdot (m + l))$ to $O(n \cdot m)$.

## 4 FET-enhanced REST Fuzzing

To manifest the utility of FET techniques, we design Leif, a FET-enhanced REST fuzzing tool, and we implement it to a command-line tool in 2,796 lines of Python code. This section elaborates the workflow of Leif, along with methodologies for collecting HTTP traffic (Section 4.1), for constructing FET lattices (Section 4.2), and for interfacing FET techniques with fuzzers (Section 4.3).

Figure 5 depicts Leif's workflow and its interaction with existing systems and tools. Leif assumes that the web service under test is already deployed on a staging server or in a production environment. The developer acquires the Leif program with a built-in FET lattice and traces HTTP traffic between the service and the clients. Then Leif identifies RESTful APIs by parsing the captured traffic and performs FET inference on parameter values. The inferred results are provided to bootstrap test case generating. Finally, Leif emits test cases and observes wrongful behaviors of the service.

**Algorithm 1:** The Forward Computation.

```
Input: A FET Lattice.
ID ← 1;  queue ← Queue(ψ⊤);
while !queue.isEmpty() do
    current ← queue.pop();
    current.ID ← ID;
    ID ← ID << 1;
    foreach ψ ⪯ current AND ψ ≠ ψ⊥ do
        queue.push(ψ);
ψ⊤.pTree ← 0;  ψ⊤.sTree ← ψ⊤.ID;
ψ⊤.tree ← ψ⊤.pTree ∨ ψ⊤.sTree;
queue ← Queue(ψ⊤);
while !queue.isEmpty() do
    current ← queue.pop();
    sTree ← 0;
    foreach ψ ⪯ current AND ψ ≠ ψ⊥ do
        sTree ← sTree ∨ ψ.ID;
    foreach ψ ⪯ current AND ψ ≠ ψ⊥ do
        ψ.pTree ← current.pTree ∨ current.ID;
        ψ.sTree ← sTree;
        ψ.tree ← pTree ∨ sTree;
        queue.push(ψ);
```

### 4.1 Collecting and Parsing HTTP Traffic

As introduced in Section 3.3, the inferred result of a parameter is contributed by its different values, and therefore the accuracy of FET inference increases when Leif witnesses more value cases. Thus developers are expected to apply suitable tracing methods. For example, monkey testing and scripted regression testing are more preferred than unit testing to collect traffic. Leif takes the HAR file (an archival format for HTTP traffic [39]), which is the standard output of network proxies (Fiddler, MitmProxy [22], etc.), and browser inspection (e.g. Chrome, and Safari). To identify parameters, the payload (including the headers, the query string, and the body) of a captured request is parsed to key-value pairs in JSON format. Due to the type collapse problem, only four data types are present: `boolean`, `number`, `string` and `object` (including `array`). Non-object-typed parameters are directly provided to FET inference while `object`-typed parameters are flattened. Since a JSON object is a tree of properties, Leif flattens it by splitting leaf properties to independent non-object-typed parameters and assigning new keys named by their JSONPaths [29], as illustrated in Figure 6. Then the flatten parameters are also provided to FET inference. Finally, FET inference receives parameters for each API where each parameter has a unique key and usually multiple values.

**Algorithm 2:** The Bitfield-boosting FET Inference.

**Input:** Parameter Values $V_n = \{v_1, \cdots, v_n\}$.
**Output:** $\psi$-$tree^n(V_n)$.

```
ψ-tree^0(V_0) ← 0;
for i ← 1 to n do
    current ← ψ_⊤;
    accepted ← true;
    while accepted do
        accepted ← false;
        foreach ψ ⪯ current do
            if ψ ∈ acceptance(v_i) then
                current ← ψ;
                accepted ← true;
    ψ-tree^i(V_i) ← ψ-tree^{i−1}(V_{i−1}) ∨ current.tree;
return ψ-tree^n(V_n);
```

### 4.2 Ubiquitous FET Lattice

**Regular Expressions for Value Formats**. In Leif's built-in ubiquitous FET lattice, value formats are specified by regular expressions. We choose to use the regular language rather than creating a new language to define value formats because it has many advantages in this scenario. Firstly, regular expressions are the de-facto descriptions of most string formats. Although regular expressions are context-free, they can still distinguish different value formats. Secondly, they are already familiar to developers, and therefore they are easy to construct without extra learning costs. Finally, to ensure the unambiguity of a FET lattice is to ensure the regular expression orthogonality of sibling FETs, which can be formally determined by finite automata [46].

**FET Lattice Constructing and Updating**. We construct the ubiquitous FET lattice by referencing popular RESTful services (e.g. Google Map, AWS, Twitter, and GitHub): (1) we crawl API documents from these services and then identify potential FETs used in these services; (2) we construct regular expressions for these FETs by referencing related RFCs (e.g. RFC3339 [35] for `ISO8601`, and RFC3986 [16] for `URI`), programming language specifications (e.g. the Java specification [34] for `PackageName`), and database schema definitions (e.g. the MongoDB data type definition [21] for `Hash`) to build a base FET lattice; (3) we apply the Bayesian regular expression generation technique [42] to discover new FETs from traffic and merge them into the base lattice; (4) we verify the unambiguity by checking the orthogonality of regular expressions for sibling FETs, using `dk.brics.automaton` library [37]. The verified lattice has 21 FETs organized in 5 levels, and we believe it is competent to model most of the RESTful services. If a developer has application-specific FETs (at the first usage or when major service updates take place), one can update the lattice by adding FETs via step (3) and repeat step (4) for unambiguity verification.

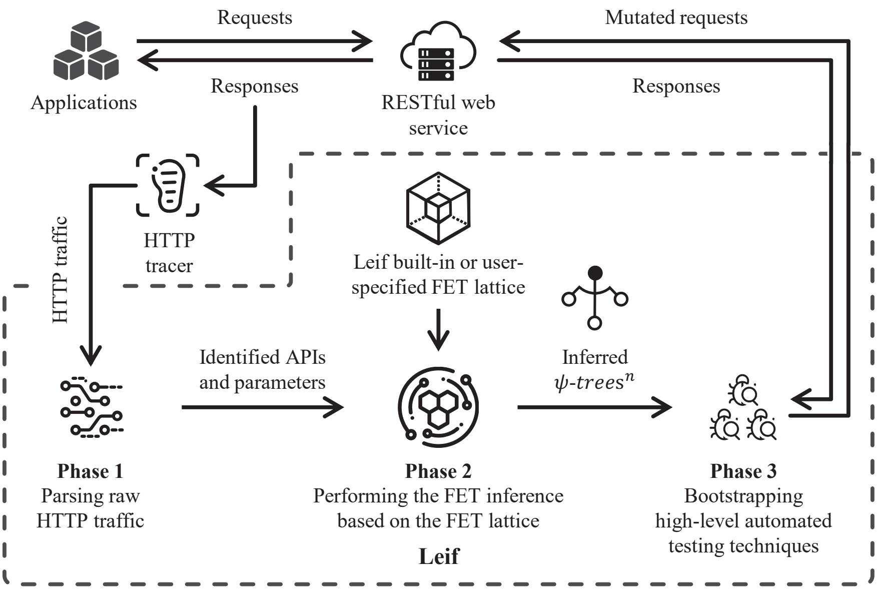

**Fig. 5.** The Workflow Architecture of Leif.

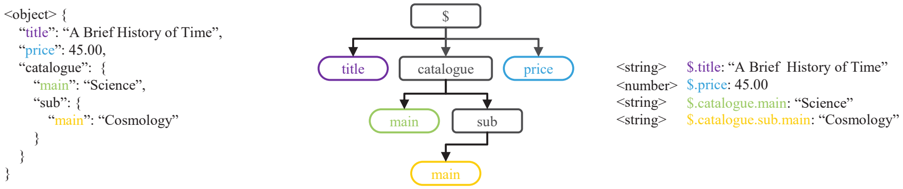

(a) The Original Parameter. (b) The Tree Structure. (c) The Flattening Result.

**Fig. 6.** An Example of Object Flattening.

**Twinning FET Inference**. We notice some parameters can be represented by multiple data types and are minimally accepted by distinct FETs in different data types. For example, an epoch datetime (elapsed seconds or milliseconds since `1970-01-01 00:00:00`) is accepted by the `EpochString` FET when it is represented by `string` while is accepted by the `Integer` FET when in `number`. Apparently, applying type casting to such parameters is very meaningful during testing. To support this feature, we implement the *twinning FET inference*. Before a value is inferred, Leif generates its twinning value if possible. If the original value is `number`-typed, Leif generates a twinning `string`-typed value (e.g. `1589809244481` $\rightarrow$ `"1589809244481"`) and vice versa (`"1589809244481"` $\rightarrow$ `1589809244481`). Then both values are inferred, and the resulting two $\psi$-*trees* are merged as if Leif witnesses two independent values. By doing so, both the `Datetime` and the `Integer` FETs are included in the final $\psi$-*tree*$^n$ of an epoch datetime parameter.

### 4.3 FET-aware Trace-driven Fuzzing

Trace-driven fuzzing tools generate test cases by replacing parameter values of captured requests with candidate values. Therefore the success of a fuzzer mainly depends on its quality of candidate values. In conventional tools, using a larger candidate dictionary is the basic strategy to increase the opportunity for triggering bugs, yet it lengthens the fuzzing time.

On the contrary, Leif provides a small but targeted dictionary for each FET and we give several examples (corresponding to Figure 3): `Number` is tried with integer overflows (8-bit, 16-bit, 32-bit, and 64-bit overflows) with signed and unsigned values; `Datetime` is tried with year overflows (`year 2038`, and `year 10,000`), invalid dates (e.g. `2019-2-29`), and timezone tweaks; `ISO8601` is tried with omitting meta characters (`"-"`, `":"`, etc.); `URI` is tried with malformed URLs (e.g. doubling `"/"`, stripping `"protocol://"`, and unescaped characters). With each parameter tagged by a $\psi$-*tree*$^n$, Leif generates test cases by exhausting dictionaries of all the FETs in the tree. Notice that, as discussed in Section 3.2, the predecessors and the siblings of the minimum acceptance describe similar but usually invalid values. Therefore, candidates from these FETs are the most likely values which can pass parameter checking and trigger bugs. For an API with multiple parameters, Leif exhausts dictionaries for one parameter each time and tests such API by iterations of exhaustion. In this way, Leif increases the opportunity to trigger bugs and meanwhile saves the fuzzing time.

## 5 Evaluation

In this section, we evaluate Leif with real-world RESTful web services, and the complete dataset of our evaluation is publicly available [19]. Specifically, we design three experiments to answer the following research questions:

**RQ-1** How accurately do FET inference results describe RESTful API parameters of complicated real-world web services?

**RQ-2** Can Leif generate effective test cases and therefore help developers to detect web service vulnerabilities in practice?

**RQ-3** Does Leif have better bug-finding capability with reduced fuzzing time when compared to existing state-of-the-art trace-driven and specification-driven fuzz testing tools?

### 5.1 FET Inference Accuracy Evaluation

In this experiment, we assume that API documents provided by the service developers are the *ground truth* and we validate the accuracy of FET inference by comparing the inferred results with the ground truth. We choose GitHub[3] and Twitter[4], and we randomly pick up 50 RESTful APIs (25 from each). We extract two pieces of information from document text: (1) parameter data types, as explicitly listed in the documents; (2) parameter value formats, as provided in the detailed descriptions (e.g. "This [the parameter `since`] is a timestamp in `ISO8601` format."[5]). We feed example requests gained from the documents to FET inference, compare the inferred FETs with the ground truth, and observe three levels of matching:

(1) **exact match**, the inferred FET is said to be an exact match if it has the exactly same data type and the value format as the ground truth;
(2) **partial match**, the inferred FET is said to be a partial match if it has the exact data type, but its value format is a proper superset of the ground truth;
(3) **mismatch**, for the remaining cases.

Intuitively, an exact match precisely describes a parameter such that a fuzzer can exploit it to generate the most targeted values. A partial match is benign, for it includes values that will not appear in practice, and a fuzzer may generate a small set of useless values based on a partial match. A mismatch indicates that the value format is not yet supported by the current FET lattice.

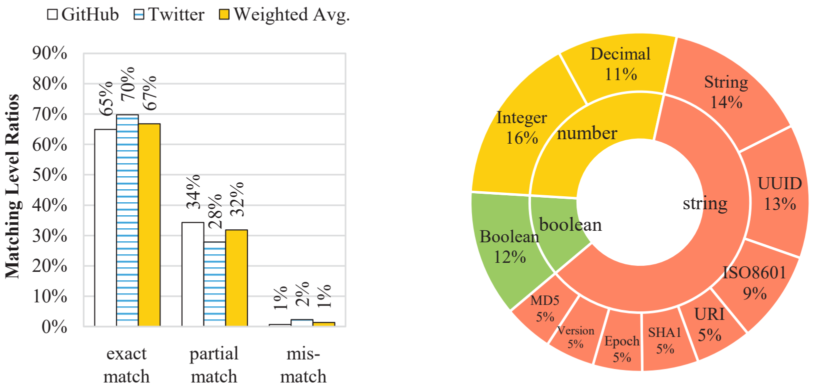

(a) FET Inference Accuracy. (b) Exact Match Distribution.

**Fig. 7.** FET Inference Accuracy Evaluation Results.

Figure 7(a) exhibits the ratios of matching on GitHub (137 parameters), Twitter (86 parameters) and the weighted average (223 parameters). In total, 149 (67%) inferred results are exact matches, and 71 (32%) are partial matches.

[3] https://docs.github.com/en/free-pro-team@latest/rest/reference
[4] https://developer.twitter.com/en/docs
[5] https://docs.github.com/en/free-pro-team@latest/rest/reference/gists

And we observe 3 mismatches in two cases: one is a binary-array parameter for file uploading and the other is an array of key-value pairs (e.g. `[["key1", "value1"], ["key2", "value2"], ...]`). Binary arrays can be supported by adding a FET (`[01]*` for the value format) to the current lattice, but Leif aims to detect logic-related bugs while binaries are usually logic-free but content-sensitive [43]. Therefore Leif simply does not mutate them. As for key-value pairs, they are actually two-dimensional arrays where the first dimension is immutable since it indicates the actual parameter key. We consider allowing developers to specify which special parameters are immutable in Leif's future version to support such cases. For the partial matches, we review the documents, and the top cases are application-specified formats such as `comma-separated strings` and `PGP signatures`. These formats are less common and developers can add application-specific FETs to their lattices by following the steps introduced in Section 4.2. Figure 7(b) exhibits the breakdown of exact matches (the inner ring is the distribution of the primitive data types and the outer ring is the inferred FETs) to quantify how FET inference improves parameter information. The coarse-grained `number`-typed (27%) and `string`-typed (61%) parameters are divided into much smaller slices (5% $\sim$ 14%). The breakdown clarifies that FET inference classifies parameters in balance, and therefore restores the collapsed types. This enables a fuzzer to generate more targeted values, which shrinks candidate space and increases the opportunity to find bugs.

### 5.2 Leif Effectiveness Evaluation

In this experiment, we select 27 popular mobile applications to evaluate the effectiveness of Leif. Each of them is backed by a commercial RESTful web service serving millions and billions of users. We monkey-test [30] each application for 20 minutes, capture HTTP traffic and run the full-stack Leif workflow. Table 1 lists the subjects and the services have an average of 133 RESTful APIs with over 19 parameters per API. We collect 46 requests per API on average which yields adequate request samples for inference. Leif reports `5XX` HTTP responses as bugs along with the corresponding traffic. We have reached out to the service owners, reported these bugs, and validated these bugs through analysis of traffic (through API URLs, parameter key-value pairs, and response data) and analysis of the involved applications (through reverse engineering and static code analysis of APKs) to eliminate any false-positive or duplicated cases. Table 2 summarizes the 11 distinct bugs found by Leif. The testing process is fully automated which mimics how developers would use Leif as a black-box fuzzing tool in practice and our following analysis mimics how to classify bugs and locate related code lines based on Leif's testing results.

**Security Bugs with Information Leakage**. Bug 1, 2 and 10 are security bugs with information leakage problems. They can be reproduced by mutating the parameter `appVer` (`VersionTag`), the parameter `platform` (`Identifier`), and the parameter `c.v` (`Integer`). These bugs not only cause service crashes but also expose sensitive information to end users (potential attackers). With the exposed information, attackers can easily design specialized attacks. For example, the

**Table 1.** Experiment Subjects of the Effectiveness Validation.

| Application Name | Category | Downloads[a] | Version | Traffic Size (MB) | # Unique APIs | # Parameters |
|---|---|---|---|---|---|---|
| Amazon | Shopping | 205M+ | 18.4.0.1 | 213 | 142 | 2,380 |
| Baidu | Tools | 2.8B+ | 11.15.0.12 | 453 | 332 | 4,742 |
| Bilibili | Video | 220M+ | 5.49.0 | 524 | 219 | 4,338 |
| Damai | Shopping | 6.6M+ | 7.6.4 | 596 | 104 | 1,535 |
| Dianping | Social | 340M+ | 10.19.12 | 629 | 148 | 2,247 |
| Eleme | Social | 180M+ | 8.26.3 | 230 | 57 | 992 |
| Hupu | Reading | 11.1M+ | 7.3.26 | 295 | 229 | 4,446 |
| iQiyi | Video | 2.5B+ | 10.10.0 | 1,338 | 257 | 7,063 |
| Jianshu | Reading | 6.4M+ | 4.16.0 | 339 | 111 | 1,609 |
| Jingdong | Shopping | 950M+ | 8.3.2 | 514 | 131 | 1,521 |
| Kaola | Shopping | 15.3M+ | 4.3.5 | 322 | 252 | 3,848 |
| Mafengwo | Trip | 21.3M+ | 9.3.33 | 340 | 151 | 3,178 |
| Meituan | Shopping | 1.4B+ | 10.3.401 | 1,111 | 58 | 1,151 |
| MissFresh | Shopping | 16.3M+ | 9.6.4 | 348 | 50 | 719 |
| ONE | Reading | 4.8M+ | 4.6.2 | 242 | 53 | 567 |
| Pinduoduo | Shopping | 1.9B+ | 4.77.0 | 795 | 79 | 866 |
| Qunar | Trip | 330M+ | 8.9.28 | 1,246 | 146 | 1,563 |
| Shanbay | Tools | 2.91M+ | 4.2.6502 | 84 | 9 | 94 |
| Sina News | News | 110M+ | 7.25.1 | 266 | 53 | 724 |
| Smzdm | Shopping | 8.5M+ | 9.5.26 | 267 | 104 | 1,866 |
| Sohu News | News | 170M+ | 6.1.8 | 591 | 201 | 3,144 |
| Tencent News | News | 2.9B+ | 5.9.00 | 1,045 | 142 | 1,796 |
| Tmall | Shopping | 310M+ | 9.1.0 | 177 | 49 | 635 |
| Toutiao | News | 2.0B+ | 7.4.8 | 1,198 | 323 | 12,408 |
| Tuniu | Trip | 79.7M+ | 10.19.0 | 217 | 68 | 772 |
| WUBA | Social | 370M+ | 9.1.2 | 79 | 123 | 5,490 |
| Xiaohongshu | Social | 66.3M+ | 6.19.0 | 295 | 20 | 334 |
| **Total** | | | | **13,754** | **3,611** | **70,028** |

[a] The statistic is from Tencent AppStore (https://sj.qq.com) up to Jan. 9th, 2020.

response data of bug 10 contains the full Java exception stack trace without any obfuscation. From the stack trace, attackers can obtain that the service uses an outdated Spring Framework[6] version which suffers from numerous security vulnerabilities [5,6,8–11]. By exploiting CVE-2020-5421 and CVE-2020-5398 [10, 11], attackers can initiate reflected file download attacks [31] to mislead users into downloading malware. And by exploiting CVE-2018-1257 [5], attackers can expose STOMP over WebSocket and then initiate denial of service attacks [17]. They can also obtain that the service uses `com.alibaba.fastjson` library[7] to deserialize user inputs. Therefore attackers can launch remote code executions by exploiting known defects in that specific library version [7,32].

Upon such cases, we suggest developers should first avoid information leakage problems by checking the service data flow, ensuring that no sensitive methods

[6] Spring Framework, https://spring.io/projects/spring-framework
[7] Fastjson, https://github.com/alibaba/fastjson

**Table 2.** Bugs Found by Leif during the Effectiveness Validation.

| Bug ID | Involved Application | Status Code | API Path | Description |
|---|---|---|---|---|
| 1 | iQiyi | 500 | /book/register | A *private* API, served for user registration. |
| 2 | Pinduoduo | 500 | /cappuccino/splash | A *private* API, served for first-screen advertising. |
| 3[a] | Sina News | 500 | /oauth2/getaid.json | A *deprecated public* API provided by Sina Weibo, served for user authorization. |
| 4[a] | Sina News | 503 | /oauth2/getaid.json | A *deprecated public* API provided by Sina Weibo, served for user authorization. |
| 5[b] | Smzdm | 502 | /integration.php | A *public* API provided by Baidu, served for inter-application integration. |
| 6[c] | Sohu News | 502 | /sendacc.jsp | A *public* API provided by 53KF, served for customer service. |
| 7[c] | Sohu News | 502 | /sendacc.jsp | A *public* API provided by 53KF, served for customer service. |
| 8 | Toutiao | 502 | /user/tab/tabs/v3 | A *private* API, probably served for inter-application redirecting. |
| 9 | Toutiao | 504 | /user/tab/tabs/v3 | A *private* API, probably served for inter-application redirecting. |
| 10 | Tuniu | 500 | /vip/recommend | A *private* API, served for content recommendation. |
| 11[b] | WUBA | 502 | /integration.php | A *public* API provided by Baidu, served for inter-application integration. |

[a] Bug 3 and bug 4 involve the same API but with different HTTP status codes.
[b] Bug 5 and bug 11 involve the same API but different applications.
[c] Bug 6 and bug 7 involve the same API path but different domain names.

(e.g., `java.lang.Exception.toString`) can be output to end users, and then diagnose security problems by analyzing server logs. Besides, they should stay alert to public vulnerability reports and timely upgrade their codebases.

**Third-party API Bugs**. We notice that 6 of the bugs involve APIs provided by third parties. Bug 3 and 4 involve the API for user authorization provided by Sina Weibo, a social networking platform serving over half a billion users. We decompile the Sina News APK and locate the related code lines. We find out the application uses a deprecated version of the API. When this API fails, an unhandled exception is propagated and causes the application to crash. It can be reproduced by injecting meta characters `"/.:/"` to the parameter `packagename` (`PackageName`) and to the parameter `mfp` (`Hash`). Bug 6 and 7 involve the API provided by a customer service platform. The application also suffers from the deprecated API and crashes when the API fails. Bug 5 and 11 are detected in different applications but involve the same API provided by Baidu. These two bugs can be reproduced by mutating the parameter `SdkVer` (`VersionTag`).

Using third-party APIs is very common, but they are often overlooked during testing. However, bugs in third-party code are as important as the application's own code, because they both mean application functionality failure to billions of end users. Our results show that Leif can find bugs across into third-party

APIs. We suggest that developers should capture application traffic and apply Leif to test untrusted third-party APIs. In addition, they should design proper exception handling logic for third-party code and timely upgrade to the latest API versions with known bugs fixed.

**Bugs with Limited Information**. We obtain very limited information from bug 8 and 9, because their responses solely contain HTTP status codes. These bugs could be as critical as the security bugs since they involve a private API and cause the service to crash. Therefore service developers can debug such APIs by following the analysis methods for the security bugs as mentioned.

### 5.3 Comparative Evaluation

**Leif vs. Trace-driven Fuzzers**. We classify Leif as a trace-driven fuzzer and we now compare it with state-of-the-art trace-driven fuzzing tools. We select BurpSuite [2], a commercial security testing fuzzer for RESTful web services, and Fuzzapi [3], an open-source general-purpose HTTP fuzzer. They provide built-in candidate dictionaries but require a series of manual configurations, including filling the URL for each API and the data type for each parameter. Therefore we only apply them to Sina News, Toutiao, and Amazon Shopping (518 unique APIs with 15,512 parameters in total). In addition, we implement NaiveFuzzer as a baseline that only understands primitive data types and randomly mutates parameter values solely based on such coarse-grained information. We construct NaiveFuzzer's candidate dictionaries by combining the dictionaries of BurpSuite and Fuzzapi.

We evaluate the bug-finding capabilities of BurpSuite, Fuzzapi, Leif, and NaiveFuzzer by comparing the number of bugs found by each tool, as reported in Figure 8(a). And we evaluate their fuzzing time by comparing the averaged number of test cases generated per parameter, as exhibited in Figure 8(b). Less generated test cases mean less test execution time, leading to the more efficient fuzzing. Considering the subjects are already well-tested before release, we believe the bug-finding capability of Leif is better than BurpSuite and Fuzzapi for Leif finds extra bugs. And NaiveFuzzer has the same capability as BurpSuite and Fuzzapi. This is because they share the same candidate space. As for fuzzing time, BurpSuite, Fuzzapi and NaiveFuzzer respectively generate $5.0\times \sim 6.7\times$, $3.6\times \sim 4.7\times$ and $6.3\times \sim 7.1\times$ test cases of Leif, indicating FET techniques bring $72\% \sim 86\%$ fuzzing time reduction.

**Leif vs. Specification-driven Fuzzers**. We now compare Leif with existing specification-driven fuzzers, which test RESTful web services based on parsing API specifications. We select RESTler [13], a state-of-the-art research fuzzer, and TnT-Fuzzer [4], an open-source robustness testing tool. They both require OpenAPI specifications [40] as input, but most of the subject services do not provide OpenAPI specifications. Therefore we construct OpenAPI specifications for Sina News, Toutiao, and Amazon Shopping by parsing HTTP traffic and referencing their official API documents.

We intend to run RESTler, but unfortunately neither the executable program nor the source code is available. According to the paper, RESTler only supports

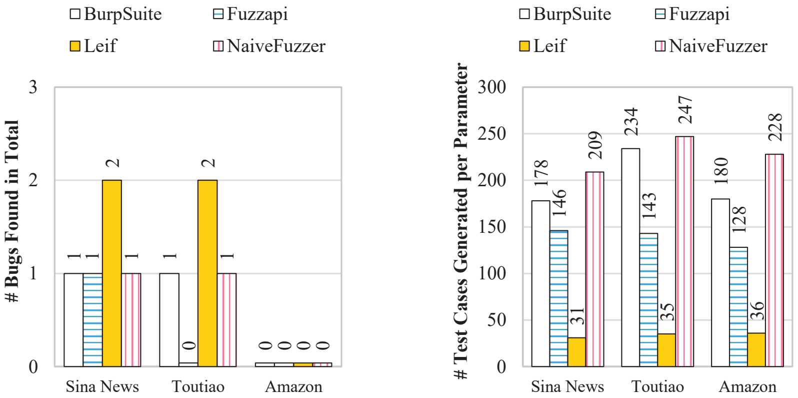

(a) Bug-finding Capabilities. (b) Fuzzing Time.

**Fig. 8.** Bug-finding Capabilities and Fuzzing Time of the Evaluated Fuzzers.

primitive data types and uses a plain candidate dictionary (consisting of `0`, `1`, `""`, and `"sampleString"`). Yet none of the bugs found by Leif can be triggered by these values, indicating that performing RESTler would fail to detect any of the bugs. And TnT-Fuzzer generates candidate values simply based on the Python `random()` function (i.e. purely random fuzzing). We configure it to generate 1,000 test cases per parameter (about 5× of NaiveFuzzer and 30× of Leif). Still, TnT-Fuzzer fails to find any bugs in the three services. We conclude that the two fuzzers' effectiveness is limited by the practical hardness of finding well-written OpenAPI specifications and the quality of their candidates. These are also the main shortcomings of all specification-driven fuzzers. Besides, many modern APIs require short-lived session tokens for access control or throttling. Specification-driven fuzzers require manual configuration or even repeated re-configuration for such parameters. In contrast, it is easy for trace-driven fuzzers to achieve this requirement by mutating freshly captured requests.

## 6 Related Work

**Model-driven Testing**. Model-driven testing [15, 26, 27, 47, 48] is usually white-box and requires using some specific modeling method (e.g. UML or DSL) through the whole lifecycle of developing, which is human-intensive and technically-limited for services across multiple servers and micro-services from different vendors. Essentially, FET techniques are also model-driven (i.e. driven by the lattice model) but only intervene in the test phase. Thus FET techniques can be practically employed to test diversified RESTful web services in black-box approaches.

**Trace-driven Fuzzing**. Trace-driven fuzzing generates test cases by mutating recorded requests. Fuzzapi [3], BurpSuite [2], AppSpider [1] and Leif all fall into this category. Existing trace-driven fuzzers mainly focus on improving the ability to capture and replay HTTP traffic. However, Leif demonstrates that FET techniques provide fundamental parameter information to fuzzers, bringing the enhanced bug-finding capability and significant fuzzing time reduction.

**Specification-driven Fuzzing**. Another main class of fuzz testing techniques is specification-driven fuzzing, such as TnT-Fuzzer [4], EvoMaster [12], and RESTler [13], which avoids the type collapse problem by assuming developers provide well-defined specifications with detailed parameter information. However, the OpenAPI [40] is the only well-established standard up to now, yet is not widely used. A survey [41] reveals that 71% developers lack the knowledge of the OpenAPI framework. Therefore, the specification-driven fuzzing is still too idealistic for testing real-world RESTful web services. In comparison, instead of asking developers for good specifications, FET techniques generate fine-grained specifications (i.e. $\psi$-$trees^n$ of parameters) on its own.

**Security Penetration Testing**. Fuzz testing techniques are also commonly purposed for security penetration testing. Commercial security penetration tools, such as BurpSuite [2], use values of SQL injections, unescaped HTML characters, XML/JSON external entities, etc., to expose system vulnerabilities. FET techniques can also be employed in security penetration testing, as demonstrated in Section 5.2. While our main goal is not limited to security testing for RESTful web services, because FET techniques improve the value selecting strategy for general-purpose REST fuzzing.

## 7 Conclusion and Future Work

In this paper, we analyze the type collapse problem and propose FET techniques to remedy this problem. As a proof-of-concept, we design and implement Leif, a FET-enhanced trace-driven fuzzing tool. We demonstrate that using FET techniques greatly improves a fuzzer's understanding of parameters, resulting in more effective fuzz testing. Our experiment results show that Leif unveils 11 new bugs in application-specific web services as well as general third-party open API platforms with 72% $\sim$ 86% fuzzing time reduction.

FET techniques are capable of effectively bootstrapping automated testing tools. We believe they are also helpful for parameter validity checking because these two technical problems are isomorphic in a sense. Thus we are beginning to study how to automatically generate or enhance parameter checking code based on FET techniques for RESTful web services.

## Acknowledgments

We would like to thank the anonymous reviewers for their valuable comments. This work was supported in part by National Key Research Development Program of China (No. 2016YFB1000502), National NSF of China (No. 61672344, 61525204, and 61732010), Shanghai Pujiang Program (No. 19PJ1430900), and Shanghai Key Laboratory of Scalable Computing and Systems.

## References

[1] AppSpider. https://www.rapid7.com/products/appspider

[2] BurpSuite. https://portswigger.net/burp

[3] Fuzzapi. https://github.com/Fuzzapi/fuzzapi

[4] TnT-Fuzzer. https://github.com/Teebytes/TnT-Fuzzer

[5] CVE-2018-1257. Available from MITRE, CVE-ID CVE-2018-1257 (Dec 6 2017), https://cve.mitre.org/cgi-bin/cvename.cgi?name=CVE-2018-1257

[6] CVE-2018-1275. Available from MITRE, CVE-ID CVE-2018-1275 (Dec 6 2017), https://cve.mitre.org/cgi-bin/cvename.cgi?name=CVE-2018-1275

[7] CVE-2017-18349. Available from MITRE, CVE-ID CVE-2017-18349 (Oct 23 2018), https://cve.mitre.org/cgi-bin/cvename.cgi?name=CVE-2017-18349

[8] CVE-2018-15756. Available from MITRE, CVE-ID CVE-2018-15756 (Aug 23 2018), https://cve.mitre.org/cgi-bin/cvename.cgi?name=CVE-2018-15756

[9] CVE-2020-5397. Available from MITRE, CVE-ID CVE-2020-5397 (Jan 3 2020), https://cve.mitre.org/cgi-bin/cvename.cgi?name=CVE-2020-5397

[10] CVE-2020-5398. Available from MITRE, CVE-ID CVE-2020-5398 (Jan 3 2020), https://cve.mitre.org/cgi-bin/cvename.cgi?name=CVE-2020-5398

[11] CVE-2020-5421. Available from MITRE, CVE-ID CVE-2020-5421 (Jan 3 2020), https://cve.mitre.org/cgi-bin/cvename.cgi?name=CVE-2020-5421

[12] Arcuri, A.: RESTful API automated test case generation with EvoMaster. ACM Trans. Softw. Eng. Methodol. **28**(1), 3:1–3:37 (2019), 10.1145/3293455

[13] Atlidakis, V., Godefroid, P., Polishchuk, M.: RESTler: Stateful REST API fuzzing. In: Atlee, J.M., Bultan, T., Whittle, J. (eds.) Proceedings of the 41st International Conference on Software Engineering, ICSE 2019, Montreal, QC, Canada, May 25-31, 2019. pp. 748–758. IEEE/ACM (2019), 10.1109/ICSE.2019.00083

[14] Aycock, J.: A brief history of just-in-time. ACM Comput. Surv. **35**(2), 97–113 (2003), 10.1145/857076.857077

[15] Baker, P., Dai, Z.R., Grabowski, J., Schieferdecker, I., Williams, C.: Model-driven Testing: Using the UML Testing Profile. Springer Science & Business Media (2007)

[16] Berners-Lee, T., Fielding, R., Masinterm, L.: RFC3986: Uniform Resource Identifier (URI): Generic Syntax. Internet Engineering Task Force (Jan 2005), https://www.rfc-editor.org/info/rfc3986

[17] Breslaw, D., Bekerman, D.: How Mirai uses STOMP protocol to launch DDoS attacks. Tech. rep., Imperva Inc. (Nov15 2016), https://www.imperva.com/blog/mirai-stomp-protocol-ddos/

[18] Chandrashekhar, R., Mardithaya, M., Thilagam, S., Saha, D.: SQL injection attack mechanisms and prevention techniques. In: International Conference on Advanced Computing, Networking and Security. pp. 524–533. Springer (2011)

[19] Chen, Y., Yang, Y., Lei, Z., Xia, M., Qi, Z.: The public dataset of Leif evaluation (Jan 2021), 10.6084/m9.figshare.12377150

[20] Chen, Y., Yang, Y., Lei, Z., Xia, M., Qi, Z.: The ubiquitous FET lattice model and verification (Jan 2021), 10.6084/m9.figshare.13622720

[21] Chodorow, K.: MongoDB: The Definitive Guide: Powerful and Scalable Data Storage. O’Reilly Media, Inc. (2013)

[22] Cortesi, A., Hils, M., Kriechbaumer, T.: MitmProxy: A free and open source interactive HTTPS proxy (2010), https://mitmproxy.org

[23] Cotroneo, D., Iannillo, A.K., Natella, R.: Evolutionary fuzzing of android OS vendor system services. Empirical Software Engineering **24**(6), 3630–3658 (2019), 10.1007/s10664-019-09725-6

[24] Cousot, P., Cousot, R.: Abstract interpretation: A unified lattice model for static analysis of programs by construction or approximation of fixpoints. In: Graham, R.M., Harrison, M.A., Sethi, R. (eds.) Conference Record of the Fourth ACM Symposium on Principles of Programming Languages, Los Angeles, California, USA, January 1977. pp. 238–252. ACM (1977), 10.1145/512950.512973

[25] Cox, N.: Directory Services: Design, Implementation and Management. Elsevier (2001)

[26] Ed-Douibi, H., Izquierdo, J.L.C., Cabot, J.: Automatic generation of test cases for REST APIs: A specification-based approach. In: 22nd IEEE International Enterprise Distributed Object Computing Conference, EDOC 2018, Stockholm, Sweden, October 16-19, 2018. pp. 181–190. IEEE Computer Society (2018), 10.1109/EDOC.2018.00031

[27] Fertig, T., Braun, P.: Model-driven testing of RESTful APIs. In: Gangemi, A., Leonardi, S., Panconesi, A. (eds.) Proceedings of the 24th International Conference on World Wide Web Companion, WWW 2015, Florence, Italy, May 18-22, 2015 - Companion Volume. pp. 1497–1502. ACM (2015), 10.1145/2740908.2743045

[28] Fielding, R.: Representational state transfer. Architectural Styles and the Design of Netowork-based Software Architecture pp. 76–85 (2000)

[29] Goessner, S.: JSONPath - XPath for JSON. http://goessner.net/articles/JsonPath p. 48 (2007)

[30] Google: Android Monkey. https://developer.android.com/studio/test/monkey

[31] Hafif, O., Spiderlabs, T.: Reflected file download: A new web attack vector. Trustwave. Retrieved March **15**, 2016 (2014), https://bit.ly/2F8YZEp

[32] Hao, M.: Fastjson 1.2.68 and earlier remote code execution vulnerability threat alert. Tech. rep., NSFOCUS, Inc. (Jun 2020), https://bit.ly/3iG0jwh

[33] Jensen, S.H., Møller, A., Thiemann, P.: Type analysis for JavaScript. In: Palsberg, J., Su, Z. (eds.) Static Analysis, 16th International Symposium, SAS 2009, Los Angeles, CA, USA, August 9-11, 2009. Proceedings. Lecture Notes in Computer Science, vol. 5673, pp. 238–255. Springer (2009), 10.1007/978-3-642-03237-0_17

[34] Joy, B., Steele, G., Gosling, J., Bracha, G.: The Java language specification (2000)

[35] Klyne, G., Newman, C.: RFC3339: Date and Time on the Internet: Timestamps. Internet Engineering Task Force (Jul 2002), https://www.rfc-editor.org/info/rfc3339

[36] Martin-Lopez, A., Segura, S., Ruiz-Cortés, A.: A catalogue of inter-parameter dependencies in RESTful web APIs. In: Yangui, S., Rodriguez, I.B., Drira, K., Tari, Z. (eds.) Service-Oriented Computing - 17th International Conference, ICSOC 2019, Toulouse, France, October 28-31, 2019, Proceedings. Lecture Notes in Computer Science, vol. 11895, pp. 399–414. Springer (2019), 10.1007/978-3-030-33702-5_31

[37] Møller, A., Bakic, A., Moran, J., et al.: Package dk.brics.automaton. Aarhus University (Jul 4 2017), https://www.brics.dk/automaton/

[38] Møller, A., Schwartzbach, M.I.: Static program analysis. Notes. Feb (2012)

[39] Morlitz, D.: HTTP archive file (May 2002), US Patent App. 09/726,985

[40] OAI (OpenAPI Initiative): The OpenAPI specification. https://github.com/OAI/OpenAPI-Specification

[41] Open API CSA Working Group: Open API survey report. Tech. rep., Cloud Security Alliance (Sep 2019), https://cloudsecurityalliance.org/blog/2019/09/11/open-api-survey-report/

[42] Ouyang, L.: Bayesian inference of regular expressions from human-generated example strings. CoRR **abs/1805.08427** (2018), http://arxiv.org/abs/1805.08427

[43] Pham, V., Böhme, M., Roychoudhury, A.: Model-based whitebox fuzzing for program binaries. In: Lo, D., Apel, S., Khurshid, S. (eds.) Proceedings of the 31st IEEE/ACM International Conference on Automated Software Engineering, ASE 2016, Singapore, September 3-7, 2016. pp. 543–553. ACM (2016), 10.1145/2970276.2970316

[44] Raychev, V., Vechev, M.T., Krause, A.: Predicting program properties from “big code”. In: Rajamani, S.K., Walker, D. (eds.) Proceedings of the 42nd Annual ACM SIGPLAN-SIGACT Symposium on Principles of Programming Languages, POPL 2015, Mumbai, India, January 15-17, 2015. pp. 111–124. ACM (2015), 10.1145/2676726.2677009

[45] Scheurer, D., Hähnle, R., Bubel, R.: A general lattice model for merging symbolic execution branches. In: Ogata, K., Lawford, M., Liu, S. (eds.) Formal Methods and Software Engineering - 18th International Conference on Formal Engineering Methods, ICFEM 2016, Tokyo, Japan, November 14-18, 2016, Proceedings. Lecture Notes in Computer Science, vol. 10009, pp. 57–73 (2016), 10.1007/978-3-319-47846-3_5

[46] Thompson, K.: Programming techniques: Regular expression search algorithm. Commun. ACM **11**(6), 419–422 (Jun 1968), 10.1145/363347.363387

[47] Vu, H., Fertig, T., Braun, P.: Towards model-driven hypermedia testing for RESTful systems. In: Majchrzak, T.A., Traverso, P., Krempels, K..H., é rie Monfort, V. (eds.) Proceedings of the 13th International Conference on Web Information Systems and Technologies, WEBIST 2017, Porto, Portugal, April 25-27, 2017. pp. 340–343. SciTePress (2017), 10.5220/0006353403400343

[48] Yuan, Q., Wu, J., Liu, C., Zhang, L.: A model driven approach toward business process test case generation. In: Liu, C., Ricca, F. (eds.) Proceedings of the 10th IEEE International Symposium on Web Systems Evolution, WSE 2010, 3-4 October 2008, Beijing, China. pp. 41–44. IEEE Computer Society (2008), 10.1109/WSE.2008.4655394

